# CaMKK2 as an emerging treatment target for bipolar disorder

**DOI:** 10.1038/s41380-023-02260-3

**Published:** 2023-09-20

**Authors:** Jacqueline Kaiser, Kevin Nay, Christopher R. Horne, Luke M. McAloon, Oliver K. Fuller, Abbey G. Muller, Douglas G. Whyte, Anthony R. Means, Ken Walder, Michael Berk, Anthony J. Hannan, James M. Murphy, Mark A. Febbraio, Andrew L. Gundlach, John W. Scott

**Affiliations:** 1 Drug Discovery Biology, Monash Institute of Pharmaceutical Sciences, Parkville, VIC 3052 Australia; 2 St Vincent’s Institute of Medical Research, Fitzroy, VIC 3065 Australia; 3 School of Behavioural and Health Sciences, Australian Catholic University, Fitzroy, VIC 3065 Australia; 4 Walter and Eliza Hall Institute of Medical Research, Parkville, VIC 3052 Australia; 5 Medicinal Chemistry, Monash Institute of Pharmaceutical Sciences, Parkville, VIC 3052 Australia; 6 Molecular and Cellular Biology, Baylor College of Medicine, Houston, TX 77030 USA; 7 The Institute for Mental and Physical Health and Clinical Translation (IMPACT), School of Medicine, Deakin University, Geelong, VIC 3220 Australia; 8 Orygen, The National Centre of Excellence in Youth Mental Health, Parkville, VIC 3052 Australia; 9 The Florey Institute of Neuroscience and Mental Health, University of Melbourne, Parkville, VIC 3052 Australia; 10 Department of Anatomy and Physiology, The University of Melbourne, Parkville, VIC 3052 Australia; 11 Department of Medical Biology, The University of Melbourne, Parkville, VIC 3052 Australia

**Keywords:** Bipolar disorder, Biochemistry

## Abstract

Current pharmacological treatments for bipolar disorder are inadequate and based on serendipitously discovered drugs often with limited efficacy, burdensome side-effects, and unclear mechanisms of action. Advances in drug development for the treatment of bipolar disorder remain incremental and have come largely from repurposing drugs used for other psychiatric conditions, a strategy that has failed to find truly revolutionary therapies, as it does not target the mood instability that characterises the condition. The lack of therapeutic innovation in the bipolar disorder field is largely due to a poor understanding of the underlying disease mechanisms and the consequent absence of validated drug targets. A compelling new treatment target is the Ca^2+^-calmodulin dependent protein kinase kinase-2 (CaMKK2) enzyme. CaMKK2 is highly enriched in brain neurons and regulates energy metabolism and neuronal processes that underpin higher order functions such as long-term memory, mood, and other affective functions. Loss-of-function polymorphisms and a rare missense mutation in human *CAMKK2* are associated with bipolar disorder, and genetic deletion of *Camkk2* in mice causes bipolar-like behaviours similar to those in patients. Furthermore, these behaviours are ameliorated by lithium, which increases CaMKK2 activity. In this review, we discuss multiple convergent lines of evidence that support targeting of CaMKK2 as a new treatment strategy for bipolar disorder.

## Introduction

Bipolar disorder is a disabling and lifelong mental condition that affects >1% of the global population that is characterised by extreme fluctuations in mood [1]. There are four subtypes of bipolar disorder that are categorised based on the occurrence, duration, and intensity of manic and depressive episodes [2]. Bipolar 1 disorder involves at least one manic episode, with or without a depressive episode or psychosis, whereas bipolar 2 disorder involves depressive episodes with a least one current or past hypomanic episode. Cyclothymic disorder is defined by recurrent subthreshold episodes of hypomania and mild depression, and unspecified bipolar disorder involves bipolar-like symptoms that do not satisfy the diagnostic criteria for the other subtypes. Patients with bipolar disorder experience significant cognitive and functional impairments and are at high risk of premature death from drug abuse and suicide, as well as comorbidities such as the metabolic syndrome and cardiovascular diseases [3–5]. Consequently, life expectancy is markedly reduced (>10 years) compared with the general population [6]. The burden of disease and high mortality rate of bipolar disorder has remained unchanged for decades, demonstrating that current standard treatments for bipolar disorder are inadequate for many patients [7].

Drug therapy remains the cornerstone treatment for bipolar disorder and is mainly based on serendipitously discovered drugs that often display limited efficacy and poor tolerability [8]. Polypharmacy involving the use of combination drug therapies is standard care in the treatment of bipolar disorder, but involves higher costs and burden, as well as increased risk of drug-to-drug interactions [9]. A large proportion of bipolar disorder patients endure residual mood symptoms, and frequent switching between mood states despite treatment, which severely impacts their lives and long-term prognosis [8]. Compounding these issues, the adverse side-effects of current drugs are associated with frequent medication changes and high rates of non-adherence to treatment [10]. These unresolved problems highlight an unmet clinical need for new drugs that are effective and safe, to achieve full remission of symptoms and better mood-stabilization [11]. The failure to develop effective, mechanism-based therapies is a direct result of major gaps in our understanding of the molecular and cellular defects that cause bipolar disorder, which has prevented the identification of rational treatment targets. Here, we review an emerging treatment target for bipolar disorder, the Ca^2+^-calmodulin-dependent protein kinase kinase-2 (CaMKK2).

## Defective Ca^2+^-signalling and bipolar disorder

Ca^2+^-signalling plays a fundamental role in the brain and regulates an array of critical functions including neuronal excitation, gene expression, neurotransmitter release, and synaptic plasticity, all of which support learning, memory and the control of mood and behaviour [12]. Defective Ca^2+^-signalling has long been implicated in the pathogenesis of bipolar disorder and related psychiatric conditions [13]. Mitochondria serve as important regulators of cellular Ca^2+^ homoeostasis and are able to modulate intracellular Ca^2+^-signalling due to their capability to absorb high levels of cytoplasmic Ca^2+^ [14]. Mitochondrial dysfunction is considered to play an underlying role in bipolar disorder, and abnormal accumulation of mitochondrial Ca^2+^ is a potent trigger of necrosis, apoptosis, and autophagy [15–18]. A recent systematic review and meta-analysis found evidence for increased free intracellular Ca^2+^ levels in patients with bipolar disorder [13]. This is supported by studies using neurons differentiated from patient-derived induced pluripotent stem cells (iPSC), which were found to display hyperexcitability and increased intracellular Ca^2+^ levels, as well as increased transcription of genes involved in Ca^2+^-signalling, compared with neurons derived from healthy unaffected controls [19, 20]. Elevated Ca^2+^-levels have also been reported in B-lymphoblasts and platelets extracted from bipolar disorder patients [21, 22]. The apparent increase in Ca^2+^ levels prompted investigations into the use of Ca^2+^-channel blockers, particularly drugs that block L-type voltage-gated Ca^2+^-channels, as potential treatments [23, 24]. However, Ca^2+^-channel blockers have failed to become an established treatment for bipolar disorder as there is mixed evidence on their efficacy, which perhaps argues that the observed increase in free intracellular Ca^2+^ in patients may simply be a marker of the underlying disturbance rather than causative [25].

An alternative view that has emerged from recent studies in mice suggest a link between reduced brain Ca^2+^ activity and bipolar disorder. For example, a two-photon imaging study of a ketamine and stress-induced mouse model of bipolar disorder, demonstrated that brain Ca^2+^ activity measured in situ was reduced in mice displaying both manic and depressive-like behaviours [26]. Similarly, conditional knockout mice lacking *Cacna1c* in the cerebral cortex, which encodes the L-type, voltage-dependent, Ca^2+^-channel, alpha 1 C subunit that is among the most commonly identified risk genes for bipolar disorder, have reduced spontaneous cortical Ca^2+^ activity and display hyperactive, manic-like behaviour [27, 28]. Likewise, loss-of-function mutations in another Ca^2+^-channel – Transient receptor potential cation channel, subfamily M, member 2 (TRPM2) – are also associated with human bipolar disorder and *Trpm2* null mice display manic-like behaviours [29].

Despite a substantial body of evidence implicating abnormalities in intracellular Ca^2+^ dynamics in the pathophysiology of bipolar disorder, the specific Ca^2+^-signalling defects in the brain that underpin the characteristic manic and depressive behaviours remains unclear.

## What is CaMKK2, and why is it a compelling treatment target for bipolar disorder?

**Fig. 1:**
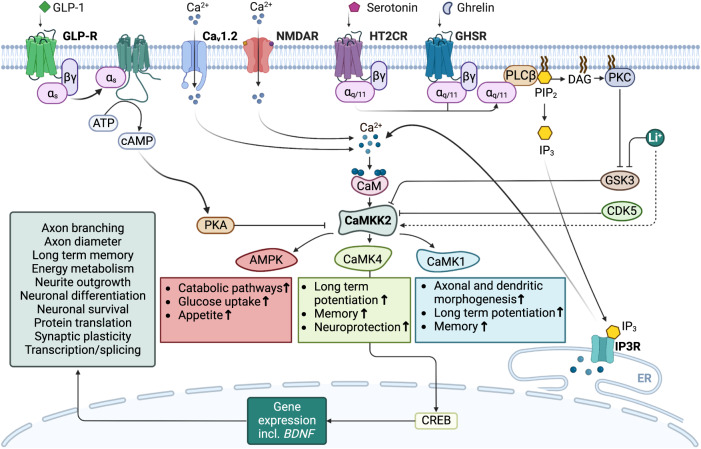
The CaMKK2 signalling pathway in the brain. CaMKK2 is activated endogenously by voltage-gated Ca^2+^-channels (Ca_v_1.2), in addition to neurotransmitter and hormone receptors that increase intracellular Ca^2+^ and cause accumulation of the Ca^2+^-calmodulin (Ca^2+^-CaM) complex. It can also be activated exogenously by the mood-stabiliser drug, lithium (Li^+^). Conversely, CaMKK2 is inhibited by CDK5 and GSK3, as well as by hormones that stimulate PKA signalling such as glucagon-like peptide-1 (GLP-1). Activated CaMKK2 directly phosphorylates three known downstream effectors (CaMK1, CaMK4 and AMPK) through which it regulates a range of neuronal and metabolic processes that support brain function.

Many neuronal processes regulated by Ca^2+^ are mediated through calmodulin, a ubiquitous Ca^2+^-sensing protein that binds and modifies the function of a diverse range of downstream effectors in response to increased intracellular Ca^2+^ [30]. CaMKK2 is a serine/threonine protein kinase and the central component of a Ca^2+^-calmodulin activated signalling pathway (Fig. 1) that regulates crucial brain functions including long-term memory formation, mood and emotional behaviour, and energy metabolism [31–33]. It is activated by voltage-dependent Ca^2+^-channels, as well as neurotransmitter and hormone receptors, that increase intracellular Ca^2+^ and cause accumulation of the Ca^2+^-calmodulin complex. These include CACNA1C, the NMDA receptor (NMDAR), and the G_αq_-protein-coupled serotonin and ghrelin receptors [34]. Conversely, hormones that stimulate cyclic-AMP-dependent protein kinase (PKA) signalling, such as glucagon-like peptide-1 (GLP-1), inhibit CaMKK2 [35]. *CAMKK2* mRNA is widely expressed in the adult human brain, with high levels in the amygdala, basal ganglia, cerebellum, cerebral cortex, hippocampus, and hypothalamus [36]. Experimental mice such as the C57BL6/6 J strain that are widely used as the genetic background for transgenic mouse models of bipolar disorder display an equivalent *Camkk2* gene expression profile in the brain [37]. The level of *CAMKK2* gene expression in human brain is low during early development, but expression levels markedly increase during late childhood/early adulthood which, notably, coincides with the average age-of-onset of bipolar disorder [38, 39].

In addition to Ca^2+^-calmodulin regulation, CaMKK2 is also regulated by phosphorylation of the S3-node, which is a control switch in the N-terminal regulatory sequence of CaMKK2 composed of three tandem serine residues (S3) that are sequentially phosphorylated by cyclin-dependent protein kinase-5 (CDK5) and glycogen synthase kinase-3 (GSK3) [40]. The S3-node functions as a two-input logic gate that inhibits CaMKK2 activity only when both the CDK5 and GSK3 signalling pathways are activated. Notably, lithium, the mood-stabiliser and frontline treatment for bipolar disorder, increases CaMKK2 activity by blocking GSK3-mediated phosphorylation of the S3-node [33]. Once activated, CaMKK2 stimulates downstream signalling pathways and gene expression programs that regulate neurogenesis, synaptic formation and plasticity, and mitochondrial function [41–44]. For example, activation of CaMKK2 in mice increases the expression of brain-derived neurotrophic factor (BDNF), a pivotal regulator of neuronal function [45]. Several meta-analyses have reported that serum BDNF levels are significantly decreased in both the manic and depressive phases of bipolar disorder [46–48]. Also, BDNF expression is increased by lithium treatment in humans and mice, and may be critical to the anti-manic effects of lithium [49, 50].

In the following sections, we expand on the molecular, genetic, pharmacological, and phenomenological evidence that link CaMKK2 to bipolar disorder.

## Upstream mechanisms that regulate CaMKK2 activity

**Fig. 2:**
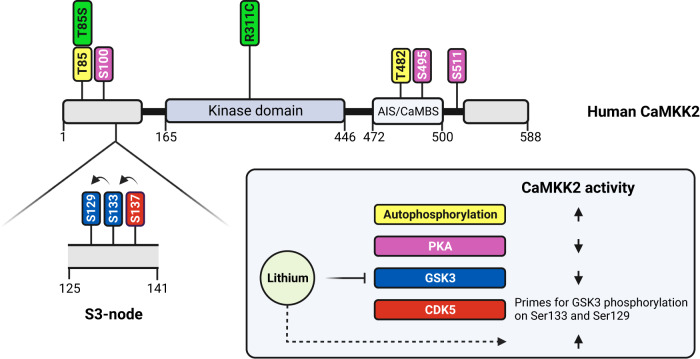
Domain structure and upstream mechanisms that regulate CaMKK2 activity. Linear schematic of the domain structure of human CaMKK2 illustrating the position of the catalytic kinase domain, the autoinhibitory (AIS) and calmodulin-binding sequences (CaMBS), and regulatory phosphorylation sites, as well as the bipolar disorder-linked T85S polymorphism and rare R311C mutation (green). Autophosphorylation of Thr85 and Thr482 (yellow) increase CaMKK2 activity. In the S3 node, phosphorylation of Ser137 (red) by CDK5 primes CaMKK2 for sequential phosphorylation on Ser133 and Ser129 (blue) by GSK3, which results in CaMKK2 inhibition. Phosphorylation of Ser100, Ser495 and Ser511 (magenta) by PKA prevents CaMKK2 activation by Ca^2+^-calmodulin, and causes binding of 14-3-3 adaptor proteins that keep CaMKK2 in an inactivated state.

The regulation of CaMKK2 activity is complex and involves an interplay between allosteric activation by Ca^2+^-calmodulin, autophosphorylation, and phosphorylation of regulatory sites by kinases that are coupled to signalling pathways controlled by various neurotransmitters and metabolic hormones [51]. CaMKK2 has a modular structure, composed of a catalytic kinase domain and a regulatory segment containing overlapping autoinhibitory and calmodulin-binding sequences, which are flanked by N- and C-terminal sequences of unknown function (Fig. 2) [52]. Binding of Ca^2+^-calmodulin to the calmodulin-binding sequence increases CaMKK2 activity by preventing the adjacent autoinhibitory sequence from hindering the catalytic site in the kinase domain [53]. In human CaMKK2, Ca^2+^-calmodulin binding induces autophosphorylation of a threonine residue (Thr85) located in the N-terminal regulatory sequence, which creates a molecular memory that enables CaMKK2 to remain in the activated state following an initial, transient Ca^2+^-signal [33]. CaMKK2 activity is also increased by autophosphorylation of another threonine residue (Thr482) located in the autoinhibitory sequence [54].

The activation of CaMKK2 by Ca^2+^-calmodulin is regulated by inhibitory crosstalk with the PKA signalling pathway [35, 55, 56]. PKA phosphorylates a conserved serine residue (Ser495) in the calmodulin-binding sequence of CaMKK2 (Fig. 2), which prevents Ca^2+^-calmodulin binding and activation. In addition, phosphorylation of two further serine residues (Ser100 and Ser511) by PKA mediates the recruitment of 14-3-3 adaptor proteins that hold CaMKK2 in an inactivated state by preventing dephosphorylation of phospho-Ser495. The death-associated protein kinase-1 (DAPK1) has also been reported to phosphorylate Ser511 on CaMKK2 [57]. Like CaMKK2, the PKA signalling pathway is also considered to play a key role in the pathogenesis of bipolar disorder, as increased cyclic AMP levels and PKA activity have been frequently observed in post-mortem brains and peripheral cells of bipolar disorder patients [58–61]. Recently, the largest whole-exome sequencing study of bipolar disorder conducted to date demonstrated a role for rare coding variations in the A-kinase anchoring protein-11 (AKAP11) as a significant risk factor in bipolar disorder aetiology [62]. AKAP proteins function as signalling hubs and are targeted to defined subcellular locations to enable spacial integration of the PKA signalling pathway with other signal transduction networks [63]. AKAP11 binds to vesicles, peroxisomes and centrosomes and forms signalling hubs at these intracellular locations with PKA, GSK3 and Ras GTPase-activating-like protein-1 (IQGAP1), all of which directly regulate CaMKK2 activity [35, 40, 64]. PKA and GSK3 are protein kinases whereas IQGAP1 is a GTPase activating protein (GAP) for the small G-proteins, CDC42 and Rac1, both of which play essential roles in neurogenesis and display altered expression in bipolar disorder [65, 66]. These data demonstrate that CaMKK2 is a component of a signalling hub that enables crosstalk between kinase and small G-protein signalling networks that regulate brain function.

CaMKK2 activity is also modulated by a Ca^2+^-calmodulin-independent mechanism involving sequential phosphorylation of three tandem serine residues in the regulatory S3-node switch (Fig. 2) [40, 54]. The S3-node modulates CaMKK2 activity by regulating the interaction between the autoinhibitory sequence and the catalytic kinase domain [67]. Phosphorylation of Ser137 in the S3-node by CDK5 primes CaMKK2 for subsequent phosphorylation on Ser133 and Ser129 by GSK3, which results in inhibition of CaMKK2 activity [40]. The CDK5 priming event is critical and functions as a gatekeeping mechanism that enables GSK3 to inhibit CaMKK2. The regulation of CaMKK2 by CDK5 and GSK3 has important implications for bipolar disorder aetiology for two reasons. First, CDK5 is a regulator of circadian rhythm, and its activity oscillates over a 24-hour period [68, 69]. This is revealing, as abnormalities in circadian rhythms are considered to play a potential underlying role in bipolar disorder, as many genes associated with bipolar disorder including *CLOCK*, *PER3* and *BMAL1* are regulated in a circadian manner [70–72]. Disruptions in circadian rhythm have also been widely reported in patients with bipolar disorder, and sleep deprivation is a known trigger, particularly of manic and hypomanic episodes [73–76]. Second, GSK3 is regulated in a circadian manner and has been linked to bipolar disorder for over two decades since the discovery that lithium, directly and indirectly, inhibits GSK3 activity [77–79]. Intriguingly, a recent study found that circadian rhythms in neurons differentiated from bipolar disorder patient-derived iPSCs predict lithium response [80]. Hyperactivation of GSK3 as a result of deficient inhibitory serine phosphorylation in the N-terminal regulatory tail occurs in bipolar disorder patients and correlates with the severity of manic and depressive symptoms [81]. GSK3 activation is also associated with oxidative stress and inflammation, both of which have been reported to be elevated in bipolar disorder patients [82–85]. Significantly, neurons from mice expressing a CaMKK2 mutant that mimics constitutive GSK3 phosphorylation of the S3-node, have neurite outgrowth abnormalities and fail to establish appropriate axon-dendrite polarity [40]. In contrast, neurons expressing a CaMKK2 mutant that mimics the effect of lithium by blocking GSK3 phosphorylation of the S3-node, display normal neurite outgrowth and polarity [33, 40]. These findings imply that signalling via the S3-node in CaMKK2 is critical for regulating neuron morphology and indicates that some of the beneficial effects of lithium on neuronal health may be mediated, at least in part, via the GSK3-CaMKK2 signalling pathway.

The integration of kinase (PKA, CDK5, GSK3) and small G-protein (IQGAP1) signalling pathways through CaMKK2, all of which have demonstrated links with human bipolar disorder, points to the possibility that bipolar disorder is a signalopathy that stems from defects in this signal transduction network, and potentially explains the clinical heterogeneity and polygenic nature of the condition.

## Downstream effector kinases of CaMKK2

Four known downstream effectors of CaMKK2 are the Ca^2+^-calmodulin-dependent protein kinases-1 and -4 (CaMK1 and CaMK4), the AMP-activated protein kinase (AMPK), and Akt/protein kinase-B (PKB) [86–93]. CaMKK2 increases the activity of each effector target by phosphorylating a highly-conserved threonine residue located within the regulatory activation loops of each of their kinase domains [51]. Through these effectors, CaMKK2 is able to regulate a variety of cellular processes that are essential for the maintenance of neuronal activity and brain function [44].

CaMK1 plays multiple roles in neuronal development and plasticity and is required for the regulation of axonal outgrowth and growth cone morphology, dendritic branching, and formation of dendritic spines and synapses [94–98]. Activation of CaMK1 promotes synaptogenesis via phosphorylation of p21-activated kinase interacting exchange factor (βPIX), which co-localises with the scaffolding proteins, G-protein-coupled receptor kinase-interacting protein-1 (GIT1) and Shank2, in the postsynaptic density of dendritic spines as part of a multiprotein complex that regulates actin dynamics [94, 99]. In hippocampal neurons, CaMK1 is required for NMDA-receptor mediated long-term potentiation, which is a form of synaptic plasticity that is considered a cellular basis of learning and memory formation [100]. CaMKK2 is also necessary for synaptic plasticity, as *Camkk2* null mice were found to have reduced long-term potentiation at the hippocampal CA1 synapse and display long-term memory impairments [42]. Several, independent meta-analyses have reported that the majority of patients with bipolar disorder experience problems with memory loss and cognition across all phases of the disorder, even during periods of remission in a manner that increases with recurrence [101–103]. While the underlying mechanisms of memory loss and cognitive impairment in bipolar disorder remain uncertain, these data indicate that defects in CaMKK2-CaMK1 signalling may play a role.

The CaMK4 signalling pathway has emerged as an important regulator of homoeostatic plasticity, which is a key process by which excitatory and inhibitory signals in neurons are actively balanced to prevent the development of hyper or hypoactivity [104]. Imbalances in neuronal activity have been implicated in a wide range of neurological disorders including bipolar disorder [105]. CaMK4 regulates two crucial aspects of homoeostatic plasticity: excitatory synaptic scaling and intrinsic excitability [104, 106]. It modulates these processes by regulating the activity of the cyclic-AMP response element-binding protein (CREB), a major regulator of neuronal gene expression and a risk gene for bipolar disorder [107]. Activation of CaMK4 in cortical neurons reduces synaptic strength and spontaneous firing rates whereas CaMK4 inhibition increases both, which suggests that CaMK4 activation generates a negative feedback signal that controls neuronal firing rates [104]. Interestingly, the anti-manic effects of lithium have been proposed to occur via a mechanism that involves synaptic scaling [108]. Like CaMK4 activation, lithium treatment has been reported to reduce synaptic strength in hippocampal neurons [49, 109].

In addition to regulating homoeostatic plasticity, the CaMKK2-CaMK4 signalling pathway also regulates cerebellar function [45]. There is an increasing recognition that the cerebellum, rather than being limited to controlling motor coordination, also regulates higher-order cognitive and emotional functions [110]. Several case studies have reported the onset of mania and rapid-cycling bipolar disorder following cerebellar lesions caused by either surgery or trauma, which indicates a potential role for the cerebellum in the pathophysiology of bipolar disorder [110–112]. This is supported by neuroimaging studies that revealed aberrant cerebellar connectivity in bipolar disorder patients experiencing psychosis [113]. Genetic deletion of either *Camkk2* or *Camk4* in mice results in decreased BDNF expression in the cerebellum, reduced cerebellar volume, as well as a decreased number of Purkinje neurons within the cerebellar cortex [45, 114, 115]. Reduced numbers of cerebellar Purkinje neurons have also been reported in patients with bipolar disorder [116]. Considering the emerging link between impaired cerebellar function and mania, and that decreased serum BDNF is a biomarker of bipolar disorder, the decrease in cerebellar BDNF expression in the *Camkk2* and *Camk4* null mice suggests that defects in CaMKK2-CaMK4 signalling within the cerebellum may be involved in triggering manic behaviour [46–49].

AMPK is an energy-sensing protein kinase and a key regulator of cellular and whole-body energy metabolism [117]. In the brain, energy metabolism is tightly regulated as neurons are dependent on glucose as their primary energy source, but are unable to store sufficient quantities of glycogen to meet demand [118]. AMPK plays a crucial role in the regulation of glucose uptake in neurons, and mediates the translocation of the glucose transporter GLUT3 to the cell surface in response to increased energy demand driven by factors such as neurotransmission [119]. Activation of AMPK in neurons also promotes mitochondrial biogenesis and oxidative glucose metabolism by increasing the expression of the master mitochondrial regulators, peroxisome proliferator-activated receptor gamma coactivator-1α (*PGC-1α*) and mitochondrial transcription factor A (*mtTFA*) [120]. This is potentially important, as there is some evidence pointing to a role for mitochondrial abnormalities in bipolar disorder although this remains controversial. For example, several studies using proton magnetic resonance spectroscopy have reported reduced levels of *N*-acetyl aspartate, a marker of impaired mitochondrial energy production, in the brains of bipolar disorder patients compared with healthy controls [121–125]. However, a systematic review and meta-analysis found this is not clear cut, as indicated by a number of other studies that failed to replicate these data [126]. CaMKK2 is essential for the activation of mitochondria-localised AMPK, and genetic deletion of either *Camkk2* or the AMPK catalytic α subunits (*Prkaa1* and *Prkaa2*) results in reduced *PGC-1α* expression and suppression of mitochondrial respiration [43, 127–130]. Lithium has been reported to increase *PGC-1α* gene expression and mitochondrial biogenesis, and to increase mitochondrial respiration; however, the mechanism is poorly understood [131, 132]. Given that lithium activates CaMKK2, it is possible that some of the lithium-induced enhancements of mitochondrial function may be partly mediated by the CaMKK2-AMPK signalling pathway. In fact, metformin, an AMPK-activating drug, and frontline treatment for type 2 diabetes, also increases *PGC-1α* expression and mitochondrial biogenesis [133]. Intriguingly, a recent randomised controlled trial found that metformin significantly improved depressive symptoms in patients with treatment-resistant bipolar depression [134]. These observations align with a pharmaco-epidemiological study that found evidence for metformin having a protective effect against the onset of mood disorders [135]. Metformin has also been shown to have an anti-depressant effect in a chronic-restraint stress model of depression in mice, which is blocked by genetic knockdown of hippocampal AMPK α catalytic subunits (*Prkaa1* and *Prkaa2*) [136]. Although there is no direct evidence at present to indicate whether metformin has anti-manic properties, these studies demonstrate that activation of the AMPK branch of the CaMKK2 signalling pathway can ameliorate depressive behaviours.

The Akt/PKB signalling pathway regulates numerous cellular processes in the brain including neurogenesis, neuronal differentiation, and synaptic plasticity [137–139]. The role of the CaMKK2-Akt/PKB signalling axis in the brain has yet to be studied; however, similar to CaMKK2, there are multiple lines of evidence that demonstrate a link between loss of Akt/PKB signalling and bipolar disorder. A recent study reported a reduction in Akt/PKB kinase activity in the dorsolateral and ventrolateral subregions of the prefrontal cortex in a large cohort of patients with bipolar disorder compared with unaffected controls [140]. Significantly, emulating this reduction in Akt/PKB signalling in the prefrontal cortex of mice resulted in cognitive impairments similar to those observed in human patients. The activation of Akt/PKB signalling has been discovered to contribute to the attenuation of manic-like behaviours in mice in response to lithium treatment [141]. This study also found that lithium responsiveness in inbred mice that differ in their response to lithium is intimately linked to the activation of Akt/PKB through the phosphorylation of Thr308. The Thr308 phosphorylation site serves as a pivotal regulatory point through which CaMKK2, as well as 3-phosphoinositide-dependent kinase-1 (PDK1), trigger the activation of Akt/PKB [87, 142]. As such, it is possible that activation of Akt/PKB signalling by lithium may involve, to some extent, activation of CaMKK2.

## Polymorphisms and a rare missense mutation that impair CaMKK2 function are associated with bipolar disorder

Whole exome sequencing and genetic association studies have uncovered a link between bipolar disorder and loss-of-function polymorphisms and mutations in human *CAMKK2*. A rare, heterozygous, de novo mutation (rs130790572; 3.98 × 10^-6^ minor allele frequency) that results in a single amino acid change (R311C) in CaMKK2 was identified from a comparative whole exome sequencing study of patients with bipolar 1 disorder and their unaffected parents [143]. The R311C missense mutation maps to a highly-conserved feature called the Histidine-Arginine-Aspartate (HRD) motif located within the catalytic kinase domain of CaMKK2 (Fig. 2). The R311C mutation results in a catastrophic loss-of-function as it destabilises key electrostatic interactions within the catalytic site that are essential for CaMKK2 activity [144]. The R311C mutant also exerts a dominant-negative effect over wild-type CaMKK2 in heterozygous carriers. Strikingly, sporadic mutation of equivalent arginine residues in the HRD motifs of unrelated protein kinases are similarly catastrophic and cause rare, monogenic forms of various human diseases [145–149]. This is highly significant, as it reveals that rare mutations in the HRD motifs of protein kinases are highly penetrant and disease-causing, which provides a strong functional rationale for the R311C mutation in the HRD motif of CaMKK2 being considered a potential monogenic model of bipolar 1 disorder.

A missense polymorphism (rs3817190; 3.83 × 10^–1^ minor allele frequency) that results in a threonine to serine (T85S) substitution at the Thr85 autophosphorylation site located within the regulatory N-terminal sequence in human CaMKK2 (Fig. 2) was shown by two independent, candidate gene association studies to display statistically significant links with bipolar and anxiety disorder [150, 151]. Like wild-type CaMKK2, autophosphorylation of the T85S variant is also triggered by Ca^2+^-calmodulin binding, but fails to keep CaMKK2 in an activated state and instead causes a temporary loss-of-activity [33].

In terms of non-coding variations, a case-control study demonstrated an association between bipolar disorder and an intronic polymorphism in *CAMKK2* (rs1063843; 1.9 × 10^-1^ minor allele frequency), where the minor allele was associated with reduced (>50%) *CAMKK2* mRNA expression in human post-mortem brains and lymphoblastoid cells [39, 152]. The rs1063843 polymorphism was also shown by functional magnetic resonance imaging (fMRI) studies to be associated with persistent activation of the left dorsolateral prefrontal cortex (DLPFC), and with increased activation of the right DLPFC and caudate nucleus during cognitive tasks that measure attentional executive control and working memory [153]. An independent fMRI study demonstrated a similar activation of the DLPFC and caudate nucleus during a working memory task in healthy volunteers given amphetamine, a psychostimulant that induces manic-like behaviours [154]. Similar to these observations in humans, methamphetamine administration in mice caused a reduction in *Camkk2* mRNA expression in the caudate nucleus [155].

Consistent with the loss-of-function effects of genetic variations in human *CAMKK2*, mice lacking *Camkk2* display behavioural traits similar to those frequently observed in bipolar disorder [33]. For example, *Camkk2* null mice are hyperactive and have deficits in pre-pulse inhibition of the startle response, both of which are seen in patients experiencing bipolar mania and psychosis, respectively [156]. *Camkk2* null mice were also found to display increased cued fear conditioning and fear-potentiated startle responses, which indicates hyperactive amygdala function [33]. Deficits in emotional processing related to amygdala hyperactivity are core features of bipolar disorder and are associated with depression and increased risk of suicide [157, 158]. These data demonstrate that genetic deletion of *Camkk2* in mice may model both manic and depressive-like behaviours.

Taken together, these findings support the view that loss-of-function genetic variations in human *CAMKK2* are prime candidates as potential underlying causes of bipolar disorder.

## Mood stabilising drugs increase CaMKK2 activity and abundance

Mood stabilisers are a class of drugs that are used to treat and manage both the manic and depressive phases of bipolar disorder [159]. Two of the most widely used drugs in this class are lithium and valproate, despite the fact that their primary targets and mechanisms of action still remain unclear. Signalling pathways and enzymes commonly affected by both lithium and valproate are more likely to be clinically relevant in bipolar disorder [160]. Therefore, identifying molecular targets that are shared between the two drugs is crucial, as it will provide some understanding of the pathogenesis of bipolar disorder, and inform the development of new, mechanism-based therapies with improved efficacy and better side-effect profiles [161].

Since the discovery of its therapeutic properties over seven decades ago, lithium remains the gold standard treatment for acute mania and prevention of recurrent bipolar disorder episodes [162, 163]. Multiple targets of lithium have been identified including inositol monophosphatase, phosphoglucomutase, and a family of four related phosphomonoesterases; however, the most convincing evidence to date suggests that GSK3 is a major effector of the therapeutic effects of lithium [79, 164, 165]. Genetic deletion of GSK3 in mice results in behaviours that mimic the mood-stabilising effects of lithium, as well as causing cognitive deficits similar to those associated with chronic lithium treatment in humans [166–169]. On the other hand, mice overexpressing GSK3 display behaviours that correlate with bipolar disorder and are desensitised to the mood-stabilising effects of lithium [170, 171]. Together, these data provide strong evidence that GSK3 is an in vivo target of lithium. GSK3 directly regulates at least forty known downstream substrates that participate in a diverse range of cellular processes [172]. The substrates that link GSK3 to bipolar disorder and lithium action are not yet clear; however, there is growing evidence that CaMKK2 could be a key effector. As described above, lithium increases CaMKK2 activity by inhibiting GSK3-mediated phosphorylation of the S3-node (Fig. 2) [33]. In addition, lithium increases CaMKK2 expression in the striatum, an area of the brain that displays structural abnormalities in bipolar disorder [173, 174]. These dual, function-enhancing effects of lithium are noteworthy, given the connection between CaMKK2 loss-of-function and bipolar disorder [33, 174]. Increased CaMKK2 activity in the brain leads to CaMK4 activation and increased expression of BDNF, which in mice is indispensable for the anti-manic actions of lithium [45, 49]. Furthermore, a recent study of genetic variants associated with lithium responsiveness in bipolar disorder patients found a significant enrichment of genes involved in glutamatergic synapse neurotransmission, which included *GSK3* and *CAMK4* [175]. Collectively, these data raise the possibility that the GSK3-CaMKK2-CaMK4 signalling axis is a principal site of action through which lithium increases BDNF expression and exerts its anti-manic effects. Also, since lithium increases both CaMKK2 activity and protein abundance, it is possible that CaMKK2 may be involved in mediating the acute as well as longer-term effects of lithium such as preventing episode recurrence [176].

Valproate was first used as an anticonvulsant to treat epilepsy but was subsequently discovered to also have mood-stabilising effects [177], which may be related to bipolar disorder and epilepsy sharing features, such as their episodic nature and being risk factors for one another [178]. Several direct targets of valproate have been discovered including the mitochondrial enzymes, succinate semialdehyde dehydrogenase and long-chain fatty acid-CoA ligase-4, as well as the family of nuclear-localised histone deacetylases that are involved in epigenetic regulation of gene expression [179–181]. However, no single target has been identified that accounts for the mood-stabilising effects of valproate. In relation to CaMKK2, a pharmacogenomic study using a methamphetamine-induced model of bipolar disorder in mice found that valproate treatment increased *Camkk2* mRNA expression in the brain caudate nucleus [155]. Intriguingly, the rs1063843 polymorphism that is associated with bipolar disorder and reduced *CAMKK2* mRNA expression in the human brain, is also associated with functional impairment of the caudate nucleus [153]. In addition to increasing *Camkk2* mRNA expression, valproate has been reported to acutely activate AMPK, which suggests that valproate may also increase CaMKK2 activity [182].

The convergent, multi-level effects of lithium and valproate on CaMKK2 activity, mRNA expression and protein abundance reveal a functional signature that indicates CaMKK2 is likely an important mediator of their mood-stabilising effects.

## CaMKK2 provides a potential link between metabolic dysfunction and bipolar disorder

Abnormalities in brain and whole-body energy metabolism are some of the most consistent features of bipolar disorder [17]. Clinically, the manic and depressive episodes of bipolar disorder closely correlate with periods of high and low energy expenditure. This is also the case phenotypically, as several studies have found objectively measured resting energy expenditure to be higher in patients with bipolar mania, and lower in patients with bipolar depression, compared with healthy controls [183–186]. Furthermore, the incidence of metabolic syndrome and type 2 diabetes is higher among patients with bipolar disorder than in the general population and is associated with a worse disease course and poor treatment outcomes [187, 188]. These discoveries have given rise to the idea that bipolar disorder is a bioenergetic and metabolic disease with psychiatric manifestations, and that disruptions in the molecular and cellular signalling networks that regulate brain energy metabolism in a biphasic manner are a primary, sufficient cause of bipolar disorder-related phenomena [189]. The energy requirements of the brain are very high due to energy intensive processes that are essential for healthy brain function, such as maintenance of ion gradients, cellular signalling, synaptic vesicle trafficking, and uptake and recycling of neurotransmitters [190]. Glucose is the major energy source, and brain activity accounts for around 25% of total body glucose consumption despite constituting just 2% of whole-body weight [191]. The disproportionate energy needs and reliance on glucose makes the brain particularly vulnerable to disruptions in the regulation of energy metabolism [192, 193].

Some of the aberrations in brain energy metabolism associated with bipolar disorder are similar to the metabolic phenotype displayed by cells lacking CaMKK2. For example, multiple neuroimaging studies have reported increased brain lactate levels in patients with bipolar disorder [194–197]. Lactate is one of the oldest and best-established biomarkers in psychiatry, first documented as early as 1934, and major psychiatric disorders have been known to be associated with acidosis since 1932 [198, 199]. Lactate is also a biomarker of bipolar disorder, as lithium treatment has been found to reverse brain lactate accumulation in patients [200]. Elevated brain lactate levels indicate a pathophysiological shift in brain energy metabolism from oxidative phosphorylation to glycolysis as the major source of energy generation. CaMKK2 dysfunction may play an underlying role in this phenomenon as genetic deletion of *Camkk2* in hippocampal neurons, myeloid cells and immortalized cell lines (HEK293 and HepG2) results in mitochondrial dysfunction that causes a similar switch in cellular energy generation from oxidative phosphorylation to glycolysis [43, 201, 202].

Another key metabolic feature of cells deficient in CaMKK2 is increased oxidative stress caused by accumulation of reactive oxygen species [202, 203]. Oxidative stress is a common hallmark of bipolar disorder and appears to increase with disease severity [82]. Indicators of oxidative stress such as lipid peroxidation have been shown to be elevated in serum from patients with bipolar disorder compared with healthy controls [204–206]. Notably, CaMKK2 suppresses lipid peroxidation by increasing the activity of NRF2, a transcription factor that promotes the expression of anti-oxidative proteins [207, 208]. Increasing NRF2 activity has been proposed as a treatment approach for bipolar disorder, and activating the CaMKK2 signalling pathway represents a potential and viable mechanism to achieve this outcome [209].

## Concluding remarks and future directions

An ever-expanding body of scientific literature points to the CaMKK2 signalling pathway as a contributing factor in the pathogenesis of bipolar disorder. Given the large clinical, social and economic burden of bipolar disorder, the development of better treatment options is an urgent priority; however, drug development for bipolar disorder remains virtually stagnant [210]. We propose CaMKK2 as a rational treatment target for bipolar disorder as it links together key facets of the condition including genetic polymorphisms/mutations, defects in signal transduction, mood stabiliser action, and metabolic dysfunction. Protein kinases are highly druggable and are the second most targeted group of drug targets after G-protein-coupled receptors [211]. At least 76 drugs that target protein kinases have been approved for clinical use, and several kinase-targeted candidate therapeutics are in various stages of preclinical and clinical development for brain-related disorders [212]. A future challenge is to identify highly selective and potent, small-molecule drugs capable of activating CaMKK2 in the brain, which can then be validated preclinically in human induced pluripotent stem cell (iPSC) and animal models of bipolar disorder. High-throughput screening of small-molecule chemical libraries using fluorescence-based kinase assays followed by hit-to-lead optimisation offers a viable strategy for identifying and developing drug activators of CaMKK2. A similar approach was successfully applied to develop selective drug activators of the protein kinase AMPK, which were revealed to bind to an unexpected allosteric site on AMPK that is distinct from the canonical AMP-binding sites [213–216]. There is also a need for high-resolution structural information on CaMKK2 to facilitate fragment and structure-based drug discovery. Like the revolution in psychiatry sparked by the discovery of the effectiveness of lithium, the development of new mechanism-based therapies with superior efficacy and tolerability hold great promise to similarly transform the treatment of bipolar disorder.
